# The Interaction between Rice Genotype and *Magnaporthe oryzae* Regulates the Assembly of Rice Root-Associated Microbiota

**DOI:** 10.1186/s12284-021-00486-9

**Published:** 2021-05-11

**Authors:** Dagang Tian, Zaijie Chen, Yan Lin, Tingmin Liang, Ziqiang Chen, Xinrui Guo, Feng Wang, Zonghua Wang

**Affiliations:** 1 State Key Laboratory of Ecological Pest Control for Fujian and Taiwan Crops, College of Life Science, Fujian Agriculture and Forestry University, Fuzhou, China; 2 Biotechnology Research Institute, Fujian Provincial Key Laboratory of Genetic Engineering for Agriculture, Fujian Academy of Agricultural Sciences, Fuzhou, 350003 China

**Keywords:** Amplicon sequencing, Microbiota, *Magnaporthe oryzae*, Rice, Meta-analysis

## Abstract

**Background:**

Utilizating the plant microbiome to enhance pathogen resistance in crop production is an emerging alternative to the use of chemical pesticides. However, the diversity and structure of the microbiota, and the assembly mechanisms of root-associated microbial communities of plants are still poorly understood.

**Results:**

We invstigated the microbiota of the root endosphere and rhizosphere soils of the rice cultivar Nipponbare (NPB) and its Piz-t-transgenic line (NPB-Piz-t) when infected with the filamentous fungus *Magnaporthe oryzae* (*M. oryzae*) isolate KJ201, using 16S rRNA and internal transcribed spacer 1 (ITS1) amplicon sequencing. The rhizosphere soils showed higher bacterial and fungal richness and diversity than the endosphere except for fungal richness in the rhizosphere soils of the mock treatment. Bacteria richness and diversity increased in the endospheric communities of NPB and Piz-t under inoculation with KJ201 (referred to as ‘NPB-KJ201’ and ‘Piz-t-KJ201’, respectively) compared with the corresponding mock treatments, with the NPB-KJ201 showing the highest diversity in the four bacterial endocompartments. In contrast, fungal richness and diversity decreased in the endospheric communities of NPB-KJ201 and Piz-t-KJ201, relative to the corresponding mock treatments, with NPB-KJ201 and Piz-t-KJ201 having the lowest richness and diversity, respectively, across the four fungal endocompartments. Principal component analysis (PCA) indicated that the microbiota of Piz-t-KJ201 of root endophytes were mostly remarkablely distinct from that of NPB-KJ201. Co-occurrence network analysis revealed that the phyla Proteobacteria and Ascomycota were the key contributors to the bacterial and fungal communities, respectively. Furthermore, a comparative metabolic analysis showed that the contents of tryptophan metabolism and indole alkaloid biosynthesis were significantly lower in the Piz-t-KJ201 plants.

**Conclusions:**

In this study, we compared the diversity, composition, and assembly of microbial communities associated with the rhizosphere soils and endosphere of Piz-t-KJ201 and NPB-KJ201. On the basis of the different compositions, diversities, and assemblies of the microbial communities among different compartments, we propose that the host genotype and inoculation pattern of *M. oryzae* played dominant roles in determining the microbial community assemblage. Further metabolomics analysis revealed that some metabolites may influence changes in bacterial communities. This study improves our understanding of the complex interactions between rice and *M. oryzae*, which could be useful in developing new strategies to improve rice resistance through the manipulation of soil microorganisms.

**Supplementary Information:**

The online version contains supplementary material available at 10.1186/s12284-021-00486-9.

## Background

Plant pathogens are an ever-increasing threat to crop production; hence, there is an urgent need to suppress disease under natural plant conditions. To achieve this, the root-associated microbiome, has been suggested as a disease-control alternative owing to its antagonistic abilities, which have been described for various soil-borne pathogens, including fungi, bacteria, oomycetes, and nematodes (Carrion et al. 2019; Cha et al. 2016; Chapelle et al., 2016; Innerebner et al. 2011; Kwak et al. 2018). Although interactions among the root endosphere, soil microbiota, and plant metabolites have the potential to dynamically affect disease outcomes (Bai et al. 2015), little is known about the real diversity of microbial communities associated with exophytic and endophytic compartments, the factors driving community assemblages, or the correlation between metabolites and the abundance of bacterial and fungal microbiomes.

The rice blast fungus *M. oryzae* (Ascomycota), a global hemibiotrophic plant fungal pathogen, causes serious blast disease at any time during rice production (Ou, 1980). Its infection is usually started in plant tissues via its germinated spores and then develops appressoria, allowing hyphae to invade the tissues and cause host cell death (Foster et al. 2016). Eevn without the formation of appressoria, several studies have demonstrated that *M.oryzae* still triggers a diverse array of immune responses in rice through pattern-recognition receptors, including altering energy metaobolism and defense-related proteins, homrone signals, ROS (Reactive Oxygen Species) generation or transcriptional reprogramming processes, and even affecting the level of the root microbiome (Cao et al. 2016; Koga et al. 2004; Marcel et al. 2010; Mallon et al. 2015; Nasir et al. 2017; Sesma and Osbourn, 2004; Yang et al. 2013).

The systemic nature of the interaction between pathogens and plant responses results in a tight linkage between leaf and root events. For example, infection of the leaves with *Epichloe coenophiala* changed the root microbial communities (Rojas et al. 2016), and whitefly infestation of pepper decreased infection by root bacterial pathogens (Yang et al. 2011). Alternatively, rhizosphere communities may affect the outcome of leaf interactions. For example, applying nematodes to the soil can reduce aphids on the leaves (Hol et al. 2013), and mycorrhizae and nitrogen-fixing bacteria in bean plants can lead to the attraction of mites (Khaitov et al. 2015). These results suggest that, after infections by phytopathogens, host plants recruit specific beneficial microbiota that enable them to resist and withstand diseases caused by these organisms (Berg et al. 2016). As *M. oryzae* is capable of infecting both the leaves and roots of rice plants, development of the rice–*M. oryzae* pathosystem would have significant implications for scientific development and disease control (Sesma and Osbourn, 2004). Moreover, this system would allow us to explore whether the metabolites involved in plant defense of the leaves are liked to root-associated microbiota.

The rice blast *R* gene *Piz-t,* a member of the *Pi2/9* multiallelic gene family, encodes a NOD-like receptor protein that specifically recognizes the *M. oryzae* effector protein AvrPiz-t (Zhou, 2016; Qu et al. 2006). Previous studies have widely utilized the NPB-KJ201 and Piz-t-KJ201 pathosystems for gene cloning, gene validation, and proteomic and transcriptional analysis (Zhou et al. 2006; Tian et al. 2016, 2018), because they exhibit high stability for susceptibility and resistance responses. Therefore, they are ideal choices for exploring the microbiome profile underlying the rice-*M. oryzae* pathosystem.

To explore the manner by which individual taxa within root-associated microbiota contribute to the interaction between rice and *M. oryzae*, we used an indoor inoculation system to minimize the possibility of natural disease factors inoculating the plant and conducted high-throughput Illumina MiSeq sequencing to structurally resolve rhizosphere and endosphere compartments. The datasets from the eight different compartments were used to identify putative microbial consortia involved in the interaction between genotype and inoculation. In addition, we also performed comparative untargeted metabolomics among the inoculated and mock treatment plants of NPB and NPB-Piz-t., we also investigated changes in metabolites and their relation to the subsequent assemblage of bacteria and fungi. The three-way interactions among root endophytic communities, rhizospheric communities, and the metabolites are key elements determining the outcome of disease.

## Results

### The Overall Microbial Communities from Root Rhizosphere Soils and Endosphere Compartments Exhibited Different Characteristics

**Fig. 1:**
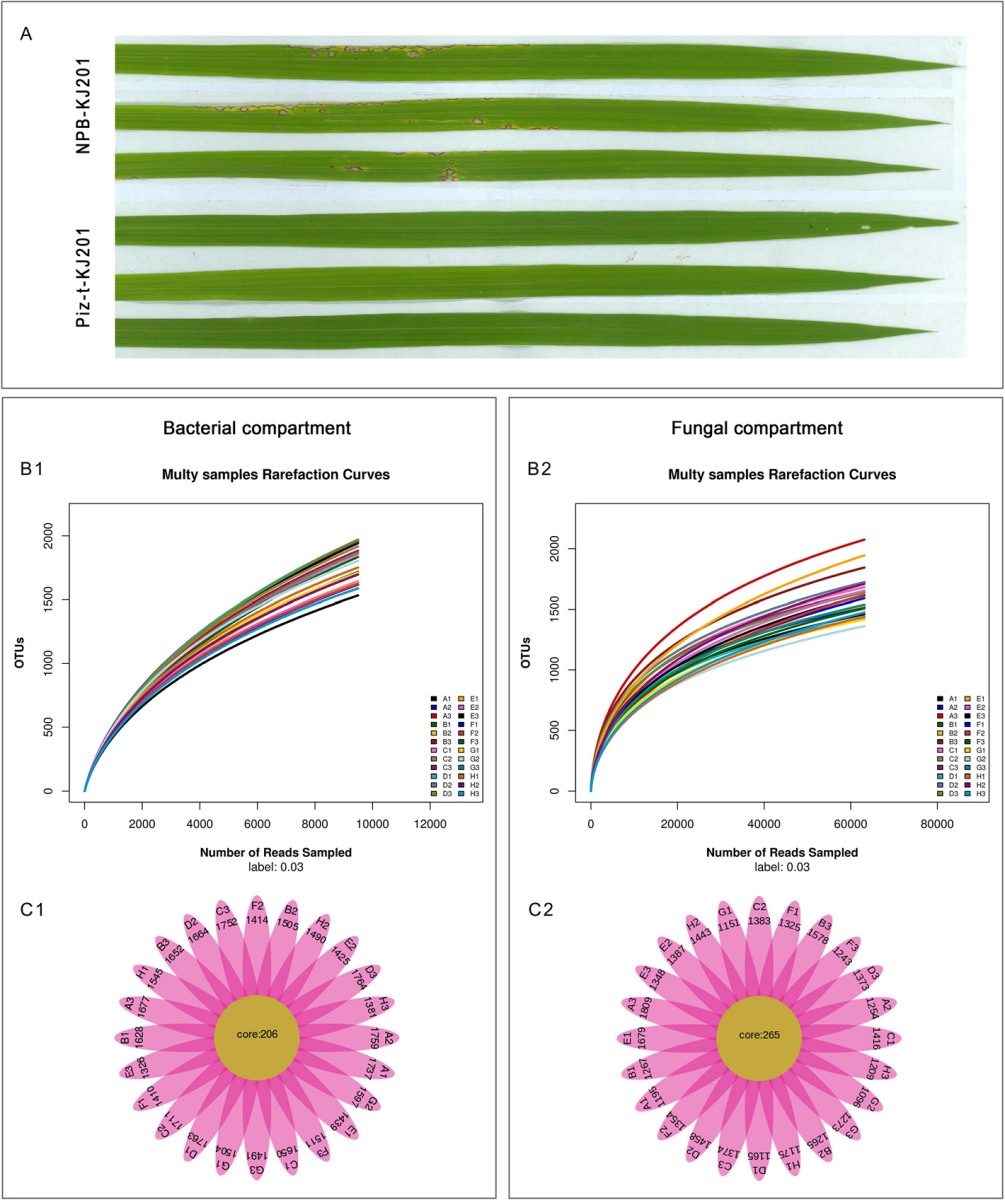
Disease phenotype of NPB-KJ201 and Piz-t- KJ201 and the data analysis of each compartment. **a** Phenotype of NPB-KJ201 and Piz-t-KJ201 at 7 days post inoculation. **b** Rarefaction curves of each samples. The rarefaction curves were constructed based on read numbers for each OTU. **c** The QTU_flowers of fungal and bacterial compartments. Each petal represents a Sample, the core number in the middle represents the number of OTU common to all samples, and the number on the petal represents the number of OTU unique to the sample. **Bacterial compartment,** A:NPB-Mock-B.R, B: Pizt-Mock-B.R, C: NPB-KJ201-B.R, D: Piz-t-KJ201-B.R, E:NPB-Mock-B.E, F: Pizt-Mock-B.E, G: NPB-KJ201-B.E, H: Piz-t-KJ201-B.E; **Fungal compartment,** A:NPB-Mock-F.R, B: Pizt-Mock-F.R, C: NPB-KJ201-F.R, D: Piz-t-KJ201-F.R, E:NPB-Mock-F.E, F: Pizt-Mock-F.E, G: NPB-KJ201-F.E, H: Piz-t-KJ201-F.E

We sampled the root-associated microbial communities when the NPB-KJ201 plants were fully diseased and analyzed the bacterial and fungal microbiota from rhizosphere soils and the endosphere (Fig. 1 a, Table 1). Microbial community composition was assessed through amplicon sequencing of the bacterial V3-V4 region and the fungal ITS1 region in the nuclear ribosomal repeat unit. To compare the diversity indices, we normalized the bacterial and fungal sequence numbers of each sample to 9, 497 and 63, 153 reads, respectively (the fewest among the 24 samples), and the numbers of operational taxonomic units (OTUs) per sample are also shown in Table 1 and Additional Table 1. The rarefaction curves suggested that the sequencing depth was sufficient to cover most of the detected species (Fig. 1 b).

**Table 1:** The eight samples and their sequencing OTUs in rhizospheric and endospheric microbiota

Bacterial Compartments	Rhizosphere				
	NPB-Mock-B.R/ A	Pizt-Mock-B.R/ B	NPB-KJ201-B.R/ C	Piz-t-KJ201-B.R/D	
	1930.33 ± 42.44	1801 ± 78.86	1910.33 ± 51.33	1936.33 ± 57.45	
	Endospere				
	NPB-Mock-B.E/E	Pizt-Mock-B.E/F	NPB-KJ201-B.E/G	Piz-t-KJ201-B.E/H	
	1602.67 ± 61.60	1651 ± 57.19	1736.67 ± 57.81	1678 ± 83.47	
Fungal compartments	Rhizosphere				
	NPB-Mock-F.R/ A	Pizt-Mock-F.R/ B	NPB-KJ201-F.R/ C	Piz-t-KJ201-F.R/D	
	1684.33 ± 338.75	1635 ± 189.14	1656 ± 22.11	1597 ± 150.74	
	Endospere				
	NPB-Mock-F.E/E	Pizt-Mock-F.E/F	NPB-KJ201-F.E/G	Piz-t-KJ201-F.E/H	
	1736.33 ± 180.90	1572.33 ± 57.57	1438.33 ± 90.59	1540.67 ± 145.91	

**Fig. 2:**
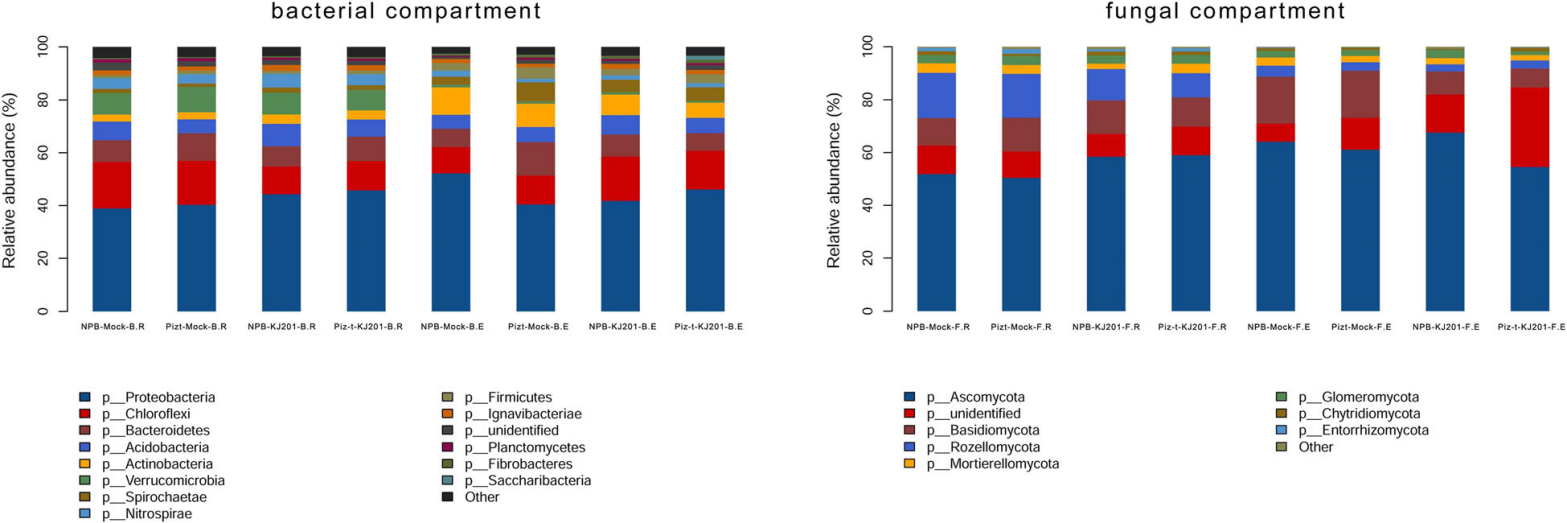
Histograms of phyla abundances in each compartment. Relative abundance plots of the fungal and bacterial taxa with greater than 1.0% of total read abundance after being pooled based on each compartment. The horizontal ordinate is the name of the sample and the ordinate is the relative abundance of the species in the sample

Overall, the bacterial and fungal cores contained 206 and 265 OTUs, respectively, which were present in all of the samples (Fig. 1C1 and C2). Analysis of these data yielded 54 and 18 taxonomic classifications at the phylum level in the bacterial and fungal communities, respectively (Additional Table 2)*.* To summarize the distribution of dominant phyla across the eight compartments, taxa ranks with over 1% relative richness across all compartments were listed. Figure 2 shows the distribution of reads and the proportion of distinct taxa in each compartment. Based on read abundance, Proteobacteria and Ascomycota were the most abundant bacterial and fungal taxa, respectively, in all of the compartments. The Piz-t-KJ201 had a higher abundance of Proteobacteria but a lower abundance of Ascomycota than NPB-KJ201(Fig. 2).

**Fig. 3:**
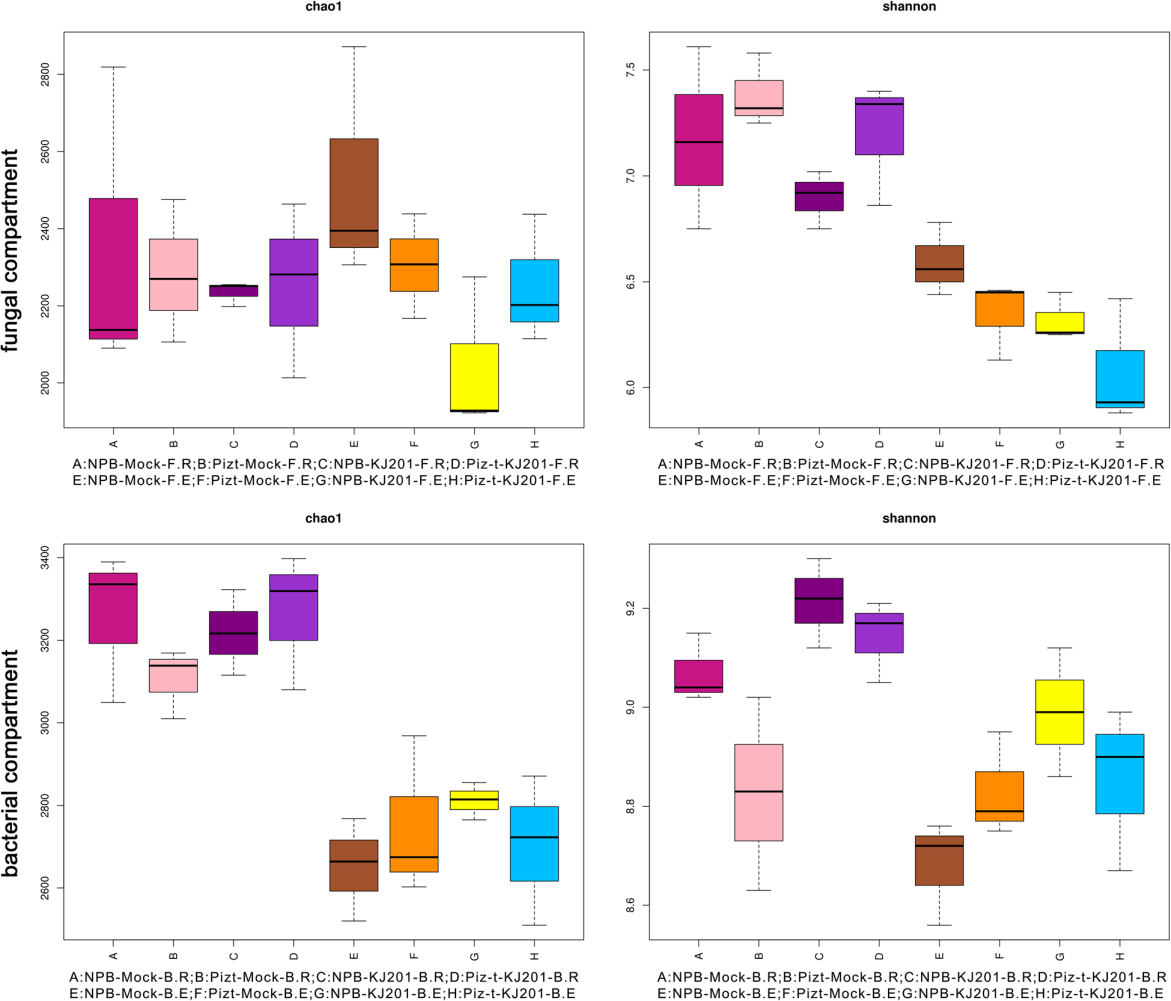
Alpha diversity analysis (ANOVA with Tukey HSD test) of eight compartments from bacterial communities and fungal communities. Rhizosphere soils and root were collected for 16 s rRNA and ITS1 sequencing, respectively. Chao1 index and Shannon index for each of the trials as determined by Alpha diversity analysis are presented. The box contains five data nodes, and the upper edge, upper quartile, middle, lower quartile, and lower edges are calculated from a set of data arranged from large to small. The horizontal ordinate is the sample/grouping name and the ordinate is the Alpha Index. The difference was of no statistic significance between each group

In general, microbial communities in the rhizosphere soils had higher diversity than those in the root endosphere. However, the rhizosphere soils of the mock treatments had lower fungal richness than the corresponding endospheric communities (Fig. 3, Additional Table 3). Further study found that bacterial richness and diversity were higher in the endospheric communities of NPB-KJ201 and Piz-t-KJ201 than the corresponding mock treatments, and that NPB-KJ201 had the highest richness and diversity in the four bacterial endocompartments. Conversely, fungal richness and diversity were lower in the endospheric communities of NPB-KJ201 and Piz-t-KJ201 compared with the corresponding mock treatments, and the endospheric communities of NPB-KJ201 and Piz-t-KJ201 had the lowest richness and diversity in the four fungal endocompartments (Fig. 3). The results were confirmed by analysis of similarities (ANOSIM) comparisons of bacterial and fungal communities among these compartments (Additional Fig. 1, Additional Table 3).

### The Differential Ascomycota OTUs Were Dominant in the Endophytes of NPB-KJ201 and the Rhizospheric Soils of Piz-T-KJ201

**Fig. 4:**
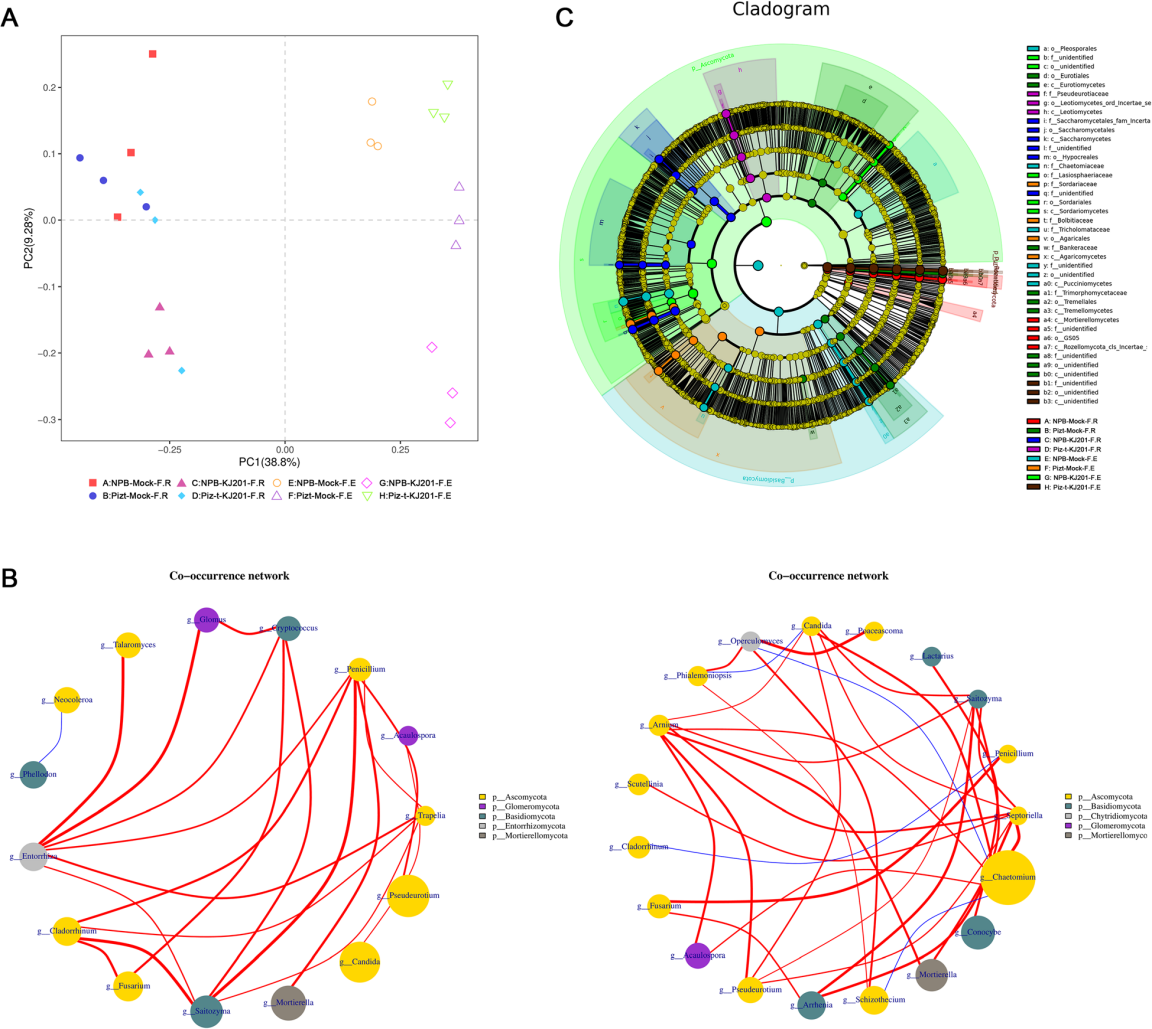
Fungal communities are separable by rhizocompartment and endocompartment. **a** Principal component analysis of OTUs abundance data indicates that the largest separation between rhizocompartment and endocompartment is spatial proximity to the root (PC1) and the second largest source of variation is host genotype and inoculation (PC2). **b** Co-occurrence networks were constructed based on correlation analysis of taxonomic profiles. Connections were drawn between nodes that were significantly (*P* < 0.01; Spearman’s rank correlation test) and highly correlated. The size and color of each node were proportional to the number of connections and module of the microbe, respectively. The colors of the links were mapping to the correlation (Red: positive correlation; Blue: negative correlation). **c** Cladogram indicating the phylogenetic distribution of fungal lineages associated with the rhizospheric and entospheric communities. The circles from inside to outside indicate fungal taxonomic levels from phylum to genus. Each small circle at each classification level represents a classification at that level, and each circle’s diameter is proportional to the taxon’s abundance. Yellow dots represent fungi not significantly varying in abundance among treatments. Biomarker fungi are coloured according to their correspondingly class colors on the right. Red nodes represent the fungal taxa that play an important role in the red group, the green nodes represent the fungal taxa that play an important role in the green group, which is applicable to the other circles. The names of the species in English letters are shown in the illustration on the right

For the fungal communities, 1260 and 1291 common OTUs were located in the Venn overlaps of different abundant OTUs across endocompartments and rhizocompartments, respectively (Additional Fig. 2). The results from PCA showed that rhizocompartments and endocompartments could be clearly separated by PC1, and the fungal endospheric community of Piz-t-KJ201 was obviously segregated by PC2 from the other three communities; PC1 and PC2 explained 38.8% and 9.28% of the relative variance, respectively (Fig. 4 a, Additional Table 4). These results demonstrated that inoculation and genotype influenced fungal communities.

There were great differences in richness and diversity among these compartments. The endospheric communities of KJ201 treatments had lower diversity and richness compared to their corresponding mock treatments, and the endospheric communities of NPB-KJ201 and Piz-t-KJ201 had the lowest richness and diversity, respectively (Fig. 2). Additionally, the network of endospheric communities (Fig. 4 b -right) was much larger, with a higher number of nodes and edges compared to the network of rhizosphere soils. The global statistics of the co-occurrence networks of the rhizosphere soils and endospheric communities are shown in Fig. 4 b. In rhizosphere soils, the major hub fungus was affiliated with the phyla Ascomycota, Glomeromycota, Basidiomycota, Entorrhizomycota, and Mortierellomycota (Fig. 4 b -left). In endospheric communities, the major hub fungus was affiliated with the phyla Ascomycota, Basidiomycota, Chytridiomycota, Glomeromycota, and Mortierellomycota (Fig. 4 b -right). Ascomycota was mainly enriched in the endospheric communities of NPB-KJ201, suggesting the multiple hinges between fungal inoculation and the host genotype. Furthermore, the rhizosphere soil communities of NPB-KJ201 and Piz-t-KJ201 were enriched by different fungal taxa of the phylum Ascomycota (Fig. 4 b). In addition, the richness of the KJ201 treatment was higher than that of the corresponding mock treatment in the rhizosphere soils, which can be attributed to some fungal spores landing on the soil and infecting rhizosphere soils during the inoculation assay.

Linear discriminant analysis effect size (LEFSE, Wilcoxon rank sum test, *p* < 0.05, LDA score > 3) was employed to identify fungal community biomarkers based on the 8 compartments of data. Seven significantly different groups of fungi were enriched in the endospheric communities of Piz-t-KJ201, including one Fungi_sp., one Schizothecium and 5 unidentified fungi (from phylum to family)(Fig. 4 c, Additional Fig. 3). In contrast, the endospheric communities of NPB-KJ201 consisted of Sordariomycetes, Sordariales, Ascomycota, Lasiosphaeriaceae, Lasiosphaeriaceae_sp, and Dothideomycetes_sp, all of which belong to the phylum Ascomycota, and four other unidentified groups (from order to genus) (Fig. 4 b, Additional Fig. 3), suggesting that the phylum Ascomycota strongly dominated fungal microbiota in the endospheric communities of NPB-KJ201.

Taken together, the above results revealed that both the host genotype and fungal inoculation determined the fungal components and assemblages of the endospheric communities, and that some Ascomycota OTUs in the endospheric communities of NPB-KJ201 may be associated with susceptibility.

### More Abundant Proteobacteria and Chloroflexi Taxa Were Separately Enriched in the Endophytes of Piz-T-KJ201 and NPB-KJ201

**Fig. 5:**
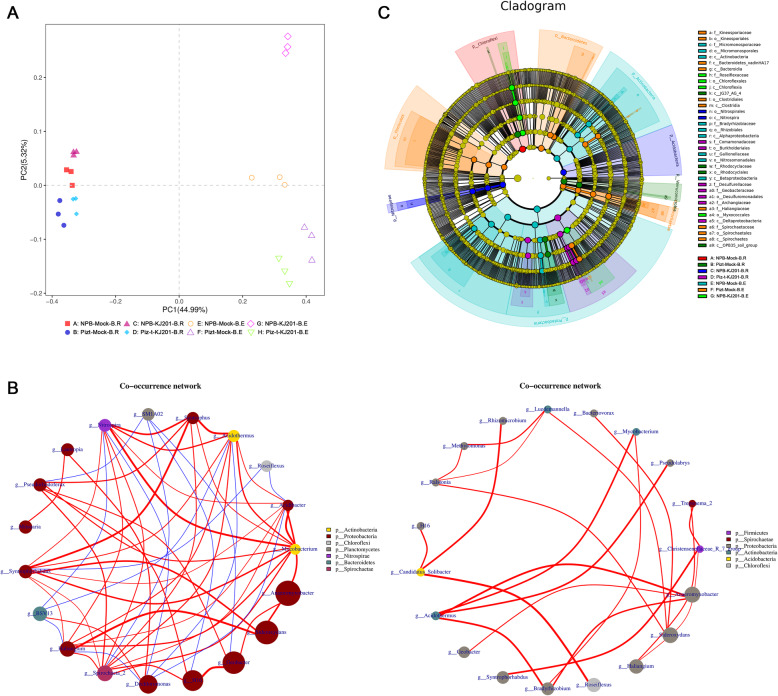
Bacterial communities are separable by rhizocompartment and endocompartment. **a** Principal component analysis of OTUs abundance data indicates that the largest separation between rhizocompartment and endocompartment is spatial proximity to the root (PC1) and the second largest source of variation is host genotype and inoculation (PC2). **b** Co-occurrence networks were constructed based on correlation analysis of taxonomic profiles. Connections were drawn between nodes that were significantly (*P* < 0.01; Spearman’s rank correlation test) and highly correlated. The size and color of each node were proportional to the number of connections and module of the microbe, respectively. The colors of the links were mapping to the correlation (Red: positive correlation; Blue: negative correlation). **c** Cladogram indicating the phylogenetic distribution of fungal lineages associated with the rhizospheric and entospheric communities. The circles from inside to outside indicate bacterial taxonomic levels from phylum to genus. Each small circle at each classification level represents a classification at that level, and each circle’s diameter is proportional to the taxon’s abundance. Yellow dots represent bacteria not significantly varying in abundance among treatments. Biomarker fungi are coloured according to their correspondingly class colors on the right. Red nodes represent thebacterial taxa that play an important role in the red group, the green nodes represent the bacterial taxa that play an important role in the green group, which is applicable to the other circles. The names of the species in English letters are shown in the illustration on the right

In addition to a comparison with fungi in those compartments, another major aim of identifying the bacterial communities was to find specialized bacterial groups that were affected by fungal inoculation. There were noteworthy overlaps in different abundant OTUs of the bacterial communities across rhizocompartments and endocompartments, with 3345 and 1288 common OTUs differentially enriched, respectively (Additional Fig. 2). Further PCA was performed to show the differences in microbial community patterns among the eight compartments (Fig. 5 a, Additional Table 4). The PCA axes in the eight compartments explained 50.31% of the total variation in the microbial community. PC1 segregated the rhizocompartments and endocompartments, and PC2 segretated each of the four groups, with 44.99%, and 5.32% of the relative variance explained, respectively (Fig. 5 a, Additional Table 4).

In contrast to the fungal communities, bacterial richness and diversity in the KJ201 treatment were higher than in the corresponding mock treatments in nearly all of the eight of the compartments; the one exception was the richness of the rhizocompartment of the NBP-KJ201, which was lower than that under the mock treatments, suggesting that the fungal inoculation enriched the bacterial communities in most of the KJ201 treatment plants, and particularly for the endospheric communities in NPB-KJ201, which had the highest richness and diversity of the four endocompartments (Fig. 3).

Next, the network of the rhizosphere soils (Fig. 5 b -left) was much larger, with a higher number of nodes and edges compared with the endospheric communities, as shown in Fig. 5 b. In rhizosphere soils, the major hub bacteria were affiliated with the phyla Actinobacteria, Proteobacteria, Chloroflexi, Planctomycetes, Nitrospirae, Bacteroidetes, and Spirochaetae (Fig. 5 b -left). In endospheric communities, the major hub bacteria were affiliated with the phyla Firmicutes, Spirochaetae, Proteobacteria, Actinobacteria, Acidobacteria and Chloroflexi (Fig. 5 b -right), from which we found that Proteobacteria was the most obviously enriched, regardless of whether they were in the rhizosphere soils or endospheric communities. Further analysis with LEFSE showed that significant Proteobacteria taxa (Wilcoxon rank sum test, *p* < 0.05, LDA score > 3) were primarily enriched by healthy plants, such as the endospheric communities of NBP-mock and the rhizosphere soils of Piz-t-KJ201, while in the diseased NPB-KJ201, the endospheric bacteria were mainly enriched by the bacterial lineages of Chloroflexi (Fig. 5 c, Additional Fig. 4). Thus, host genotype and fungal inoculation drove the variables for bacterial community assemblage, and the dominant Proteobacteria and Chloroflexi taxa may potentially be associated with the development of disease.

### Tryptophan Metabolism Is Associated with Microbial Differences

**Fig. 6:**
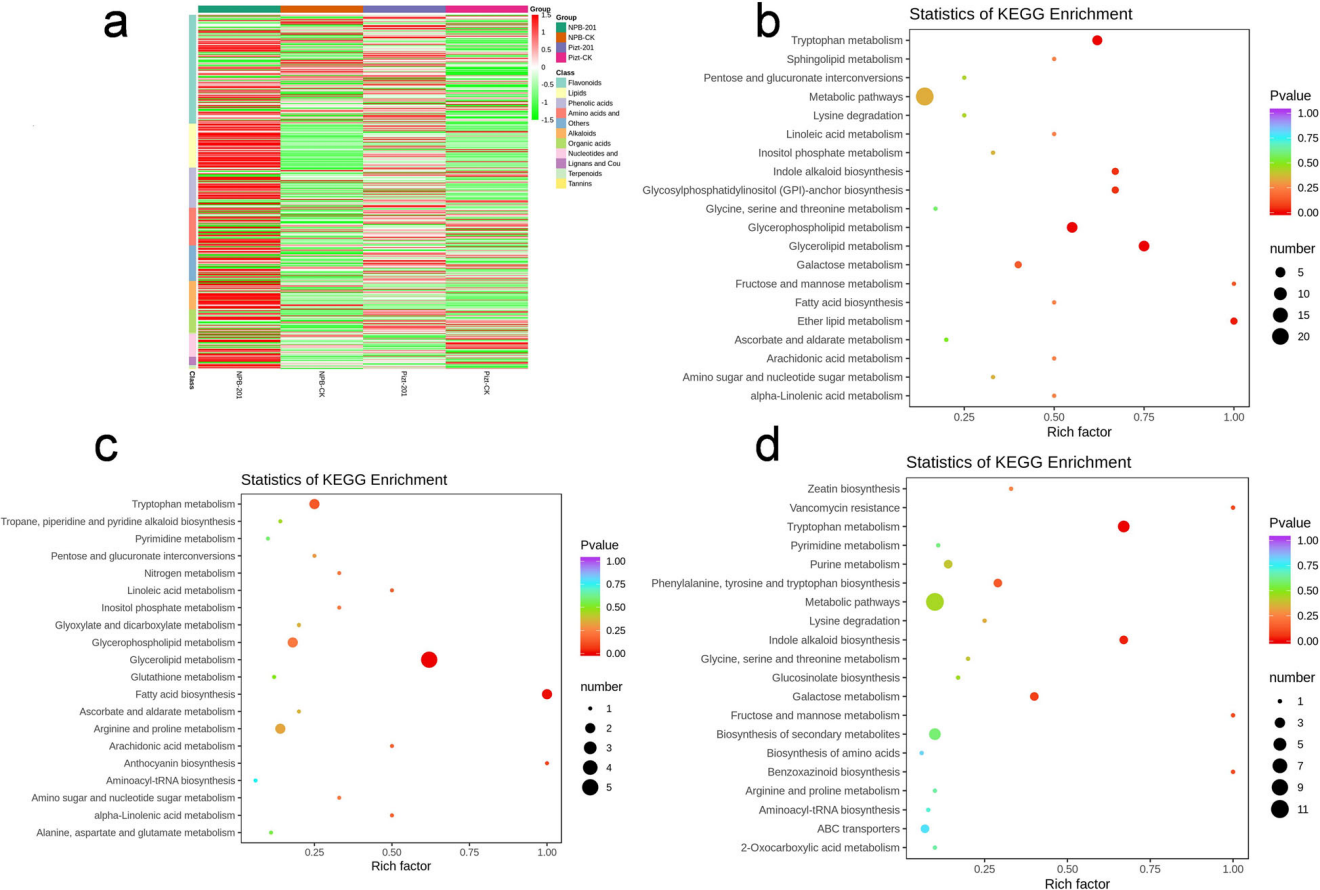
Clustering and KEGG analysis of the defferentially expressed metabolisms between Piz-t-KJ201, NPB-KJ201, and their mock treated. **a** Heatmap showing the relative abundance of functional categories of the four groups. b,c,d KEGG enrichment of differentially abundance metabolisms specific to comparison NPB-KJ201/NPB-Mock, Piz-t-KJ201/Pizt-Mock, and Piz-t-KJ201/NPB-KJ201, respectively. The Rich factor is the ratio of differentially expressed metabolites numbers annotated in this pathway term to all metabolites numbers annotated in this pathway term

Metabolites can act as key substrates or signaling molecules that affect microbial composition (Hu et al. 2018), and endophytes can also produce or consume diverse classes of plant-associated secondary metabolites (Jain and Pundir, 2017). Therefore, it was necessary to test the hypothesis that the composition and concentrations of metabolites differed between Piz-t-KJ201 and NPB-KJ201. The results revealed that there were 78 and 52 differentially expressed metabolites (DEMs) in Piz-t-KJ201 and NPB-KJ201, compared to the mock treatments, respectively, among which 22 were shared (Fig. 6 a, Additional Table 5).

Among the shared DEMs, both N-cis-sinapoyltyramine and N-cis-feruloyltyramine were the most significantly downregulated compounds in Piz-t-KJ201 compared to NPB-KJ201, with fold change values of 0.000045 and 0.000055, respectively (*P* < 0.05). Additional KEGG enrichment analysis showed that metabolic pathways, biosynthesis of secondary metabolism, glycerophospholipid metabolism, glycerolipid metabolism, and tryptophan metabolism were the most significant pathways in NPB-KJ201 compared with mock treatments (Fig. 6 b, Additional Table 6), while metabolic pathways, fatty acid biosynthesis, and tryptophan metabolism were the most significant suppressed pathways in Piz-t-KJ201 compared with mock treatments (Fig. 6 c, Additional Table 6). Furthermore, in comparing Piz-t-KJ201 to NPB-KJ201, the most significant pathways were tryptophan metabolism and indole alkaloid biosynthesis (Fig. 6 d, Additional Table 6).

A previous study demonstrated that tryptophan metabolism was influenced by the compositional background of the bacteria, and that many key enzymes such as tryptophan hydroxylase, were involved in bacterial catalysis and utilization of the neurohormonal products of the host (Taj and Jamil, 2018), which was highlighted by the lower accumulation of N-cis-sinapoyltyramine and N-cis-feruloyltyramine in Piz-t-KJ201 compared to NPB-KJ201 in this study. Consequently, these metabolites may influence bacterial communities that contribute to the host immune response.

## Discussion

It is widely recognized that many factors shape the microbial communities of the rhizosphere and endosphere of plant roots (Mendes et al. 2011; Lumibao et al. 2020). The growth environment, soil composition, and host play major roles; however, the extent to which each affects the final outcome remains unclear (Edwards et al. 2015). To characterize the microbial compositions of the rhizosphere and endosphere, and to gain insights into the effects of fungal inoculation and host genotype on each of the two compartments, we utilized two rice varieties (NBP and NBP-Piz-t) that upon KJ201 and mock inoculations to demonstrate the difference in microbial communities in the rhizosphere and endosphere of NPB and NPB-Piz-t.

By using alpha diversity analysis, our results revealed that bacterial richness and diversity were higher in the rhizosphere soil community than in the endophytic community (Fig. 3). Earlier studies indicate that invasion of the soil microbiome follows a two-step selection model; first the rhizosphere is colonized and then the roots are invaded (Bulgarelli et al. 2013; Edwards et al. 2015; Zarraonaindia et al. 2015; Eyre et al. 2019). However, microbial entry into rice roots is not a passive process, and plants may select certain microbial consortia to fill the root colonizing niche. This conclusion is supported by our observation that the relative abundance of the phylum Proteobacteria was increased in the endosphere compared with the rhizosphere soil of Piz-t-KJ201, and that the relative abundances of Actinobacteria and Bacteroidetes were decreased in the rhizosphere soil relative to the endosphere (Fig. 2). Surprisingly, the richness of endosphere fungi in the NPB-mock and Piz-t-mock treatments was higher than in their corresponding rhizosphere soil compartments (Fig. 3). This phenomenon was similar to prior findings that may be derived from stochastic processes (Gottel et al. 2011; He et al. 2020).

The primary aim of this study was to identify core microbiome members for the future development of agricultural products. Previous studies on the plant-associated microbiome have identified large differences between healthy and diseased individuals (Trivedi et al. 2012). However, whether these differences are the cause or consequence of disease development remains unclear. Although it is difficult to disentangle the potential drivers behind this, there must be some key microbes for rice resistance and diseased plants that are affected by genotype, environment, or stress (Edwards et al., 2015; Zhalnina et al. 2018; Zhong et al. 2019). To assess the major members that comprise the microbiomes, we clustered microbes with more than 1% of the total reads based on the abundance of phyla in each compartment. Our results revealed that the phyla Ascomycota and Proteobacteria were the most abundant within the fungal and bacterial communities, respectively, across the eight compartments. A further co-occurrence network analysis also supported this finding.

Typically, Ascomycota dominates the fungal community in rice paddy fields (Jiang et al. 2016; Yuan et al. 2018), and some members of this phylun are rice pathogens, such as *M. oryzae* (*Pyricularia oryzae*), the causal agent of rice blast (Chaibub et al. 2016). In general, the sexual phase *Pyricularia oryzae* has a heterothallic mating system determined by a single master locus that carries either a Mat1–1 or Mat1–2 sequences, which leads to a high degree of variability in disease development by *M. oryzae* isolates (Bardwell, 2005; Kang et al., 1994). Therefore, maybe the coexistence of different mating lineages in the same susceptible plant may be one of the major reasons for the increased abundance of Ascomycota taxa in the endosphere of NPB-KJ201. Likewise, Proteobacteria dominate the soil bacterial community (Hussain et al. 2011; Jiang et al. 2016) and include important bacteria that degrade a wide range of metabolites for microbial turnover (Vu et al. 2006). For example, many proteobacterial rhizosphere isolates respond to N-acyl homoserine lactone quorum-sensing signals that act as interkingdom signals that influence plant gene expression, induce systemic plant resistance and affect plant growth and development (Venturi and Fuqua, 2013). In our study, fungal inoculation induced the plants to recruit beneficial microbes from the soil, such as some proteobacterial lineages, to increase resistance, especially in the resistant plants, which further mediated plant systemic resistance to deleterious fungal colonization, as shown in Fig. 3. Thus the respective abundances of the phylum Proteobacteria suggest that the rhizosphere and endosphere of rice plants tend to resist adverse external factors by becoming enriched with bacteria that are beneficial for growth and health. Taken together, these results imply that inoculation with *M. oryzae* and the host genotype predetermine the future of microbial composition and structure, as previously described (Bulgarelli et al. 2012; Bouffaud et al. 2014; Lebeis et al. 2015;Xu et al. 2020; Zhong et al. 2019).

Recently, Seybold et al. (2020) found that bacterial diversity in leaves increased in a cultivar that was susceptible to *Zymoseptoria tritici,* a hemibiotrophic plant pathogen, compared with a resistant cultivar, during the biotrophic stage of infection. They suggested that defense-related metabolites and systemic acquired resistance might be involved in the decrease in bacterial diversity in the resistant cultivar. Our results are generally consistent with those of a previous study; the bacterial communities of NPB-KJ201 were more diverse than those of Piz-t-KJ201 (Fig. 3).

By comparing the metabolic components of Piz-t-KJ201 and NPB-KJ201, with the mock treatments, we identified a variety of immune-related and antimicrobial metabolites differentially produced among them. Tryptophan (Trp) metabolism acts as a common hub for the biosynthesis of many immune-related compounds (Alkhalaf and Ryan, 2015). On the one hand, many of these metabolites are well known for their antioxidant properties and support cell wall reinforcement after pathogen infection (Ishihara et al. 2008). Alternatively, Trp can be incorporated by bacteria into a large array of bioactive natural products that act as antifungal compounds (Alkhalaf and Ryan, 2015). Therefore, Trp-derived metabolites may also systemically influence the composition and structure of microbes. In our study, along with the differential accumulation of some Trp-derived metabolites such as N-cis-sinapoyltyramine and N-cis-feruloyltyramine, we observed an obvious difference in the microbial endosphere communities of the KJ201 treatments (Fig. 2), in which Proteobacteria and Chloroflexi were increased in Piz-t-KJ201 and NBP-KJ201, respectively. However, fungal richness was correspondingly reduced (Fig. 3). Experimental validation in future studies will confirm the direct or indirect role of Trp-derived metabolites on microbial community composition and structure.

Based on all of these results, we propose a hypothetical model for the whole infection process. Initially, fast activation of defense-related metabolites and systemic acquired resistance would have occurred in response to KJ201 treatment. Subsequently, some bacterial growth would benefit from the Trp-derived metabolites, which would lead to an increase in endosphere bacterial communities that feed on those metabolites and a decrease in endosphere fungal diversity and richness in both NPB-KJ201 and Piz-t-KJ201 treatments compared with their corresponding mock treatments.

Many unidentified taxa were enriched in the endophytes of Piz-t-KJ201 and rhizosphere soils of NPB-KJ201 (Fig. 4), a finding that was consistent with previous studies that also identified a substantial proportion of unclassified sequences (Edwards et al. 2015). These unknown members may be due to gaps between genetic knowledge and old species descriptions (Tedersoo et al. 2018). Fortunately, a protocol for high-throughput bacterial isolation from root samples has been established, and a growing number of new updated online dabtabases are available for the best nomenclature and identification of microbial species, which facilitates the identification of unknown taxa by their functional traits (Prakash et al. 2017; Zhang et al. 2021).

Although our results have potential significance in managing diseases by modulating the composition of soil microbiota, further experiments are needed to confirm the functional and ecological roles of some microbes, especially for those unidentified communities over time and space, and to understand the interaction of the metabolites with microbial communities. Therefore, comprehensive combined approaches are required to expand our understanding of microbial communities and meet agricultural needs.

## Conclusion

In this study, we provide a complete survey of the bacterial and fungal rhizospheric and endospheric microbiota in both Piz-t-KJ201 and NPB-KJ201. The NPB-KJ201 plants had the highest endospheric bacterial diversity and richness across the four bacterial endocompartments but had the lowest endospheric fungal richness across the eight endocompartments. In contrast, the Piz-t-KJ201 plants possessed distinct and greater endospheric bacterial diversity and richness than the corresponding Piz-t-mock plants but had the lowest abundance of endospheric fungal diversity across the eight endocompartments. In addition, statistically significant enrichment of the phyla Proteobacterial and Ascomycota occurred in the endospheric communities of Piz-t-KJ201 and NPB-KJ201, respectively. Further comparative metabolomics analysis between Piz-t-KJ201 and NPB-KJ201 strengthened that some metabolites may involved in those bacterial community changes. Taken these results together demonstrate that the proposed role of the rice genotype and inoculation in determining the composition and assembly of root-associated microbial communities.

## Methods

### Materials and Methods

#### Soil Collection, Plant Materials and Blast Isolates

The soil was collected from a single rice field site at the same moment in Fuzhou, Fujian, China (E119°18′, N26°05′). The rice cultivars NPB and NPB-Piz-t were used in this experiment, and NPB-Pizt was generated as described in our previous study (Tian et al. 2018). Rice seedlings were grown approximately 2 ~ 3 weeks to 3–4 leaves in a pot filled with local soil that contained a microbial community structure that retained natural conditions. The plants were inoculated by spraying at a concentration of 5 × 10^5^ spores/ml, and a mock treatment served as the control. After inoculation, the seedlings were maintained in the dark for 24 h at approximately 28 °C and then maintained at humidity of more than 95% to favour disease development.

#### Sample Collection

Soil samples and roots were collected at seven days post-inoculation when NPB-KJ201 plants were obviously diseased (Fig. 1 a). Rhizosphere soils were sampled using a root shaking method as previous described (Inceoğlu et al. 2010), The roots and above-ground parts were segregated for endosphere microbiota and metabolomics analyses, respectively. The soil remaining attached on the roots was considered to be rhizosphere soil and the remaining roots were defined as the endosphere compartment. After the harvested roots were surface sterilized, the surfaces of these roots were rubbed onto Luria-Bertani (LB) plates for incubating overnight at 30 °C, and those that showed no microbial growth were used for the further experiment. Three biological replicates for each of these lines were collected for each inoculation treatment. In total, 12 root endosphere samples and 12 rhizosphere soil samples were collected for 16 s rRNA and ITS1 amplicon sequencing. The above-ground samples were collected for a metabolomics analysis.

#### 16S rRNA and ITS1 Gene Amplicon Sequencing

Total DNA was extracted from soil using a DNA Kit (Omega Bio-tek, Norcross, GA, USA) according to the manufacturer’s instructions. The DNA quality and concentration were monitored using a NanoDrop 1000 spectrophotometer (Thermo Scientific, Waltham, MA, USA). The total DNA was used as PCR templates, and 16S amplicon libraries were generated using the PCR primers 319F (5′-CCTACGGGNGGCWGCAG-3′) and 806R (5′-GGACTACHVGGGTWTCTAA T-3′) with an adapter (index) that targets the V3 and V4 variable regions of bacterial/archaeal 16S rRNA genes (Walters et al. 2015; Eyre et al. 2019). Strongly amplified products were chosen for additional experiments. ITS1 amplicon libraries were generated using the PCR primers ITS1F (5′-CTTGGTCATTTAGAGGAAG.

TAA-3′) and ITS1R (5′-GCTGC GTTCTTCATCGATGC-3′) (Gardes and Bruns, 1993; White et al. 1990; Usyk et al., 2017). PCR amplifications were conducted using Phusion® High-Fidelity PCR Master Mix with GC Buffer (New England Biolabs, Ipswich, MA, USA), and PCR products were detected using 2.0% agarose gels. Target bands were purified using a QIAquick Gel Extraction Kit (Qiagen, Hilden, Germany). The PCR products were used for 16S rRNA, and the ITS1 sequencing was conducted at the Novogene Institute (Beijing, China) using the MiSeq platform. Briefly, DNA was fractionated by ultrasound. Sequencing libraries were then prepared with Illumina’s instructions, and an Illumina MiSeq platformc (2017) followed by HiSeq2000 (2016).

#### Data Processing

OTUs and microbial diversity analyses were conducted as previously described (Edwards et al. 2015; Zhong et al., 2019). The standard operating procedure of QIIME (V1.7.0) was employed to filter the sequencing quality for each forward and reverse fastq file. The sequences were demultiplexed into each sample based on whether they were derived from bacterial or fungal sequences, and then paired-end sequences of each sample were trimmed for their quality and length using Trimmomatic (V0.36) (Bolger et al., 2014), yielding 1,862,933 and 2,459,716 high quality reads for the 16S V3-V4 and ITS1datasets, respectively. Finally, operational taxonomic units (OTUs) were picked using the script pick_otus.py of QIIME via the UCLUST method at a similarity cutoff of 97%. Taxonomic classification of the representative sequence for each OTU was done using QIIME’s version of the Ribosomal Database Project’s classifier against the Greengenes 16S rRNA and Unite ITS database (13_5 release) using default parameters. Chloroplast, mitochondrial, and unclassified reads were discarded. The representative sequences for each OTU were aligned using PyNAST in QIIME. All samples were randomly rarefied to the lowest number of sequences (9497 and 63,153 sequences for the 16S V3-V4 and ITS1datasets, respectively) for further analysis.

Venn diagrams of the taxonomic assignment were constructed using the VennDiagram V1.6.20 package (Chen and Boutros 2011). Rarefaction curves of Chao1 and Shannon index were analysed by using Perl scripts in QIIME. Weighted and Unweighted UniFrac distrance were calculated from normalized OTU tables for Beta Diversity analysis, PCA utilizing the Weighted and Unweighted UniFrac distrances were calculated using the R package Ape. ANOSIM analysis was carried out using R (version: 3.4.3) and the Vegan package in R (version: 2.3.0). A permutation testing (999 permutations) was performed to validate the fitness of ANOSIM models. LEfSE analysis was performed using the online LEfSE programme based on a normalized OTU table. For LEfSE analysis, the Kruskal–Wallis rank sum test was employed to test for significantly different species within groups at an alpha value of 0.05 and a threshold of 3. Co-occurrence analyses were carried out using the Python module ‘SparCC’ and network visualizations were constructed using Cytoscape (v. 3.4.0) and Gephi (Shannon et al. 2003).

#### Metabolomics Measurement and Analysis

The freeze-dried above ground parts were crushed using a mixer mill (MM 400, Retsch technology, Haan, Germany) with zirconia beads for 1.5 min at 30 Hz. A total of 100 mg powder was weighed and extracted overnight at 4 °C with 0.6 ml 70% aqueous methanol. Following centrifugation at 10,000 g for 10 min, the extracts were absorbed (CNWBOND Carbon-GCB SPE Cartridge, 250 mg, 3 ml; ANPEL, Shanghai, China, www.anpel.com.cn/cnw) and filtered (SCAA-104, 0.22 μm pore size; ANPEL, Shanghai, China, http://www.anpel.com.cn/) for UPLC-MS/MS analysis.

The hierarchical cluster analysis (HCA) results of samples and metabolites were presented as heatmaps with dendrograms. HCA was conducted using an R package p heatmap (Qin et al. 2019). KEGG enrichment analysis identified metabolites that were annotated using the KEGG Compound database (http://www.kegg.jp/kegg /compound/), and the annotated metabolites were then mapped to the KEGG Pathway database (http://www.kegg.jp/ kegg/pathway.html).

## Supplementary Information

**Additional file 1: Fig. S1.** ANOSIM analysis was performed based on a Bray-Curtis distance matrix from each compartment to calculate the differences between rhizosphere soils and endosphere compartments. Permutation test, number of permutation is 999. **Bacterial communities,** A:NPB-Mock-B.R, B: Pizt-Mock-B.R, C: NPB-KJ201-B.R, D: Piz-t-KJ201-B.R, E:NPB-Mock-B.E, F: Pizt-Mock-B.E, G: NPB-KJ201-B.E, H: Piz-t-KJ201-B.E; **Fungal communities,** A:NPB-Mock-F.R, B: Pizt-Mock-F.R, C: NPB-KJ201-F.R, D: Piz-t-KJ201-F.R, E:NPB-Mock-F.E, F: Pizt-Mock-F.E, G: NPB-KJ201-F.E, H: Piz-t-KJ201-F.E.

**Additional file 2: Fig. S2.** Venn map of bacterial and fungal communities in the rhizospheres soils and endosphere of NPB-KJ201 and Piz-t-KJ201 plants. **Bacterial communities,** A:NPB-Mock-B.R, B: Pizt-Mock-B.R, C: NPB-KJ201-B.R, D: Piz-t-KJ201-B.R, E:NPB-Mock-B.E, F: Pizt-Mock-B.E, G: NPB-KJ201-B.E, H: Piz-t-KJ201-B.E; **Fungal communities,** A:NPB-Mock-F.R, B: Pizt-Mock-F.R, C: NPB-KJ201-F.R, D: Piz-t-KJ201-F.R, E:NPB-Mock-F.E, F: Pizt-Mock-F.E, G: NPB-KJ201-F.E, H: Piz-t-KJ201-F.E.

**Additional file 3: Fig. S3.** Indicator fungal groups across 8 compartments with LDA values higher than 3.LDA: linear discriminant analysis. A:NPB-Mock-F.R, B: Pizt-Mock-F.R, C: NPB-KJ201-F.R, D: Piz-t-KJ201-F.R; E:NPB-Mock-F.E, F: Pizt-Mock-F.E, G: NPB-KJ201-F.E, H: Piz-t-KJ201-F.E.

**Additional file 4: Fig. S4.** Indicator bacterial groups across 8 compartments with LDA values higher than 3. LDA: linear discriminant analysis. A:NPB-Mock-B.R, B: Pizt-Mock-B.R, C: NPB-KJ201-B.R, D: Piz-t-KJ201-B.R; E:NPB-Mock-B.E, F: Pizt-Mock-B.E, G: NPB-KJ201-B.E, H: Piz-t-KJ201-B.E.

**Additional file 5: Table S1.** OTUs for bacterial and fungal communities.

**Additional file 6: Table S2.** Topphylum for bacterial and fungal taxa across 8 compartments.

**Additional file 7: Table S3.** Statistical table of alpha diversity index for bacterial and fungal communities.

**Additional file 8: Table S4.** PCA for bacterial and fungal compartments.

**Additional file 9: Table 5.** The significance difference metabolism of Piz-t-KJ201 and NPB-KJ201.

**Additional file 10: Table S6.** The statistic analysis of KEGG enrichment for each group.

## Data Availability

The sequencing data have been submitted to the Sequence Read Archive (SRA) database (https://www.ncbi.nlm.nih.gov/sra) under the accession number PRJNA674417. All data generated or analysed during this study are included in this published article [and its supplementary information files.

## Abbreviations

DEMs: Differentially expressed metabolites
HCA: Hierarchical cluster analysis
ITS1: Internal transcribed spacer 1
KEGG: Kyoto encyclopedia of genes and genomes
LEFSE: Linear discriminant analysis effect size
LDA: Linear discriminant analysis
OTUs: Operational taxonomic units
PCA: Principal component analysis
Piz-t-KJ201: NPB-Piz-t inoculated with KJ201
NPB: Nipponbare
NPB-Piz-t: NPB Piz-t-transgenic line
NPB-KJ201: NPB inoculated with KJ201
