# Supplementary figures and images for: The Interaction between Rice Genotype and Magnaporthe oryzae Regulates the Assembly of Rice Root-Associated Microbiota
Source: Rice (N Y). 2021 May 11;14:40. doi: 10.1186/s12284-021-00486-9 (PMC8113375; doi:10.1186/s12284-021-00486-9)

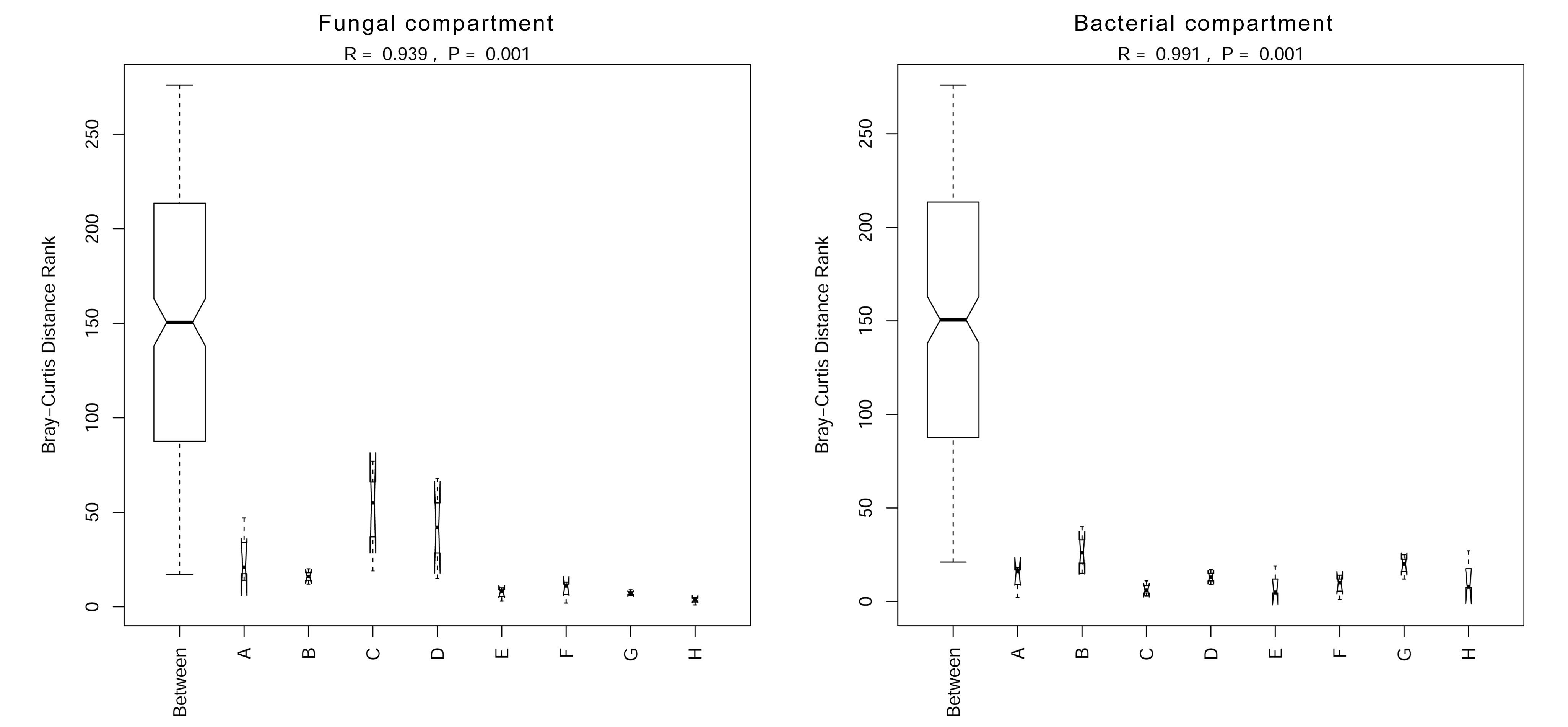

Supplement: Supplementary file 1 — Additional file 1: Fig. S1. ANOSIM analysis was performed based on a Bray-Curtis distance matrix from each compartment to calculate the differences between rhizosphere soils and endosphere compartments. Permutation test, number of permutation is 999. Bacterial communities, A:NPB-Mock-B.R, B: Pizt-Mock-B.R, C: NPB-KJ201-B.R, D: Piz-t-KJ201-B.R, E:NPB-Mock-B.E, F: Pizt-Mock-B.E, G: NPB-KJ201-B.E, H: Piz-t-KJ201-B.E; Fungal communities, A:NPB-Mock-F.R, B: Pizt-Mock-F.R, C: NPB-KJ201-F.R, D: Piz-t-KJ201-F.R, E:NPB-Mock-F.E, F: Pizt-Mock-F.E, G: NPB-KJ201-F.E, H: Piz-t-KJ201-F.E. [file 12284_2021_486_MOESM1_ESM.tif]

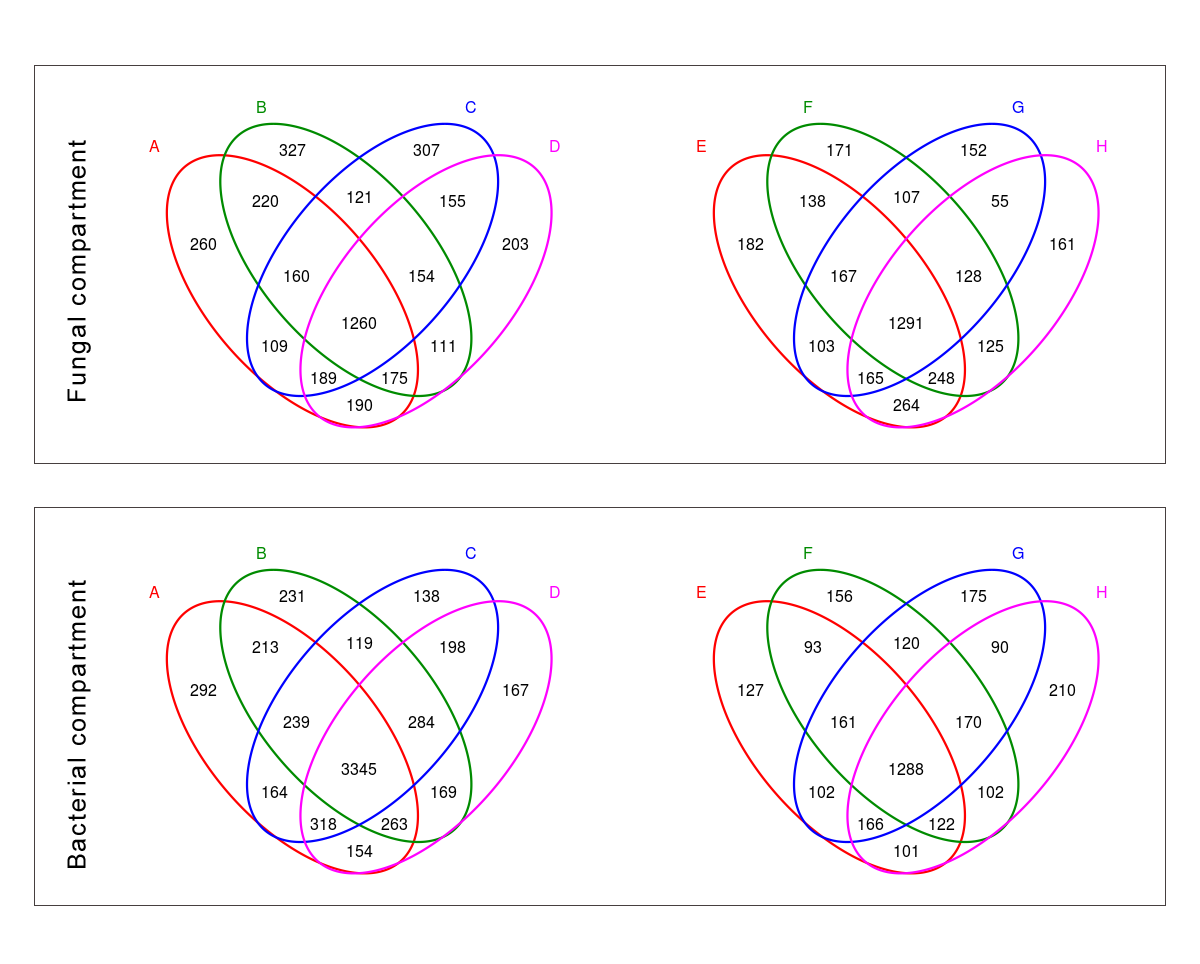

Supplement: Supplementary file 2 — Additional file 2: Fig. S2. Venn map of bacterial and fungal communities in the rhizospheres soils and endosphere of NPB-KJ201 and Piz-t-KJ201 plants. Bacterial communities, A:NPB-Mock-B.R, B: Pizt-Mock-B.R, C: NPB-KJ201-B.R, D: Piz-t-KJ201-B.R, E:NPB-Mock-B.E, F: Pizt-Mock-B.E, G: NPB-KJ201-B.E, H: Piz-t-KJ201-B.E; Fungal communities, A:NPB-Mock-F.R, B: Pizt-Mock-F.R, C: NPB-KJ201-F.R, D: Piz-t-KJ201-F.R, E:NPB-Mock-F.E, F: Pizt-Mock-F.E, G: NPB-KJ201-F.E, H: Piz-t-KJ201-F.E. [file 12284_2021_486_MOESM2_ESM.tif]

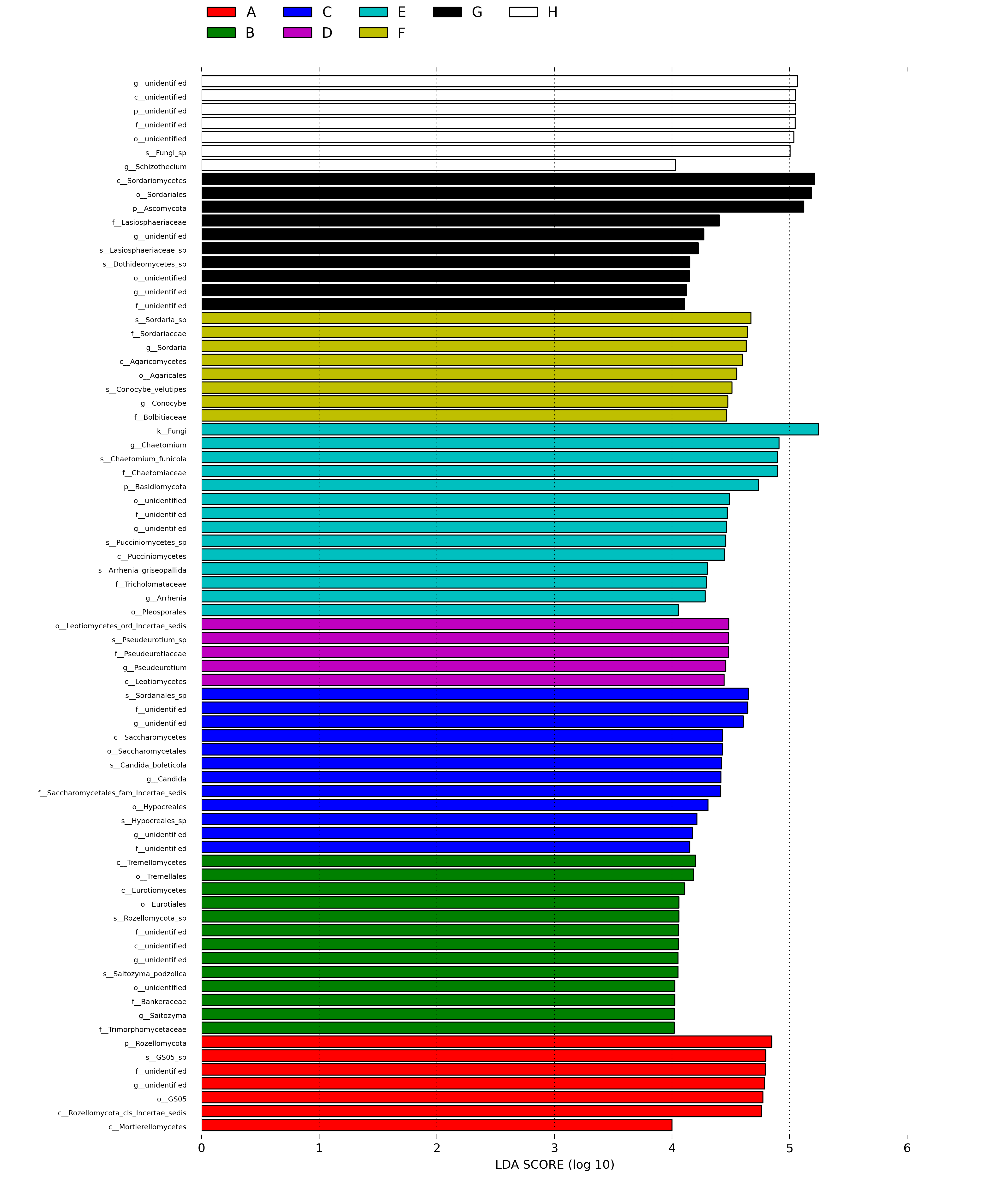

Supplement: Supplementary file 3 — Additional file 3: Fig. S3. Indicator fungal groups across 8 compartments with LDA values higher than 3.LDA: linear discriminant analysis. A:NPB-Mock-F.R, B: Pizt-Mock-F.R, C: NPB-KJ201-F.R, D: Piz-t-KJ201-F.R; E:NPB-Mock-F.E, F: Pizt-Mock-F.E, G: NPB-KJ201-F.E, H: Piz-t-KJ201-F.E. [file 12284_2021_486_MOESM3_ESM.png]

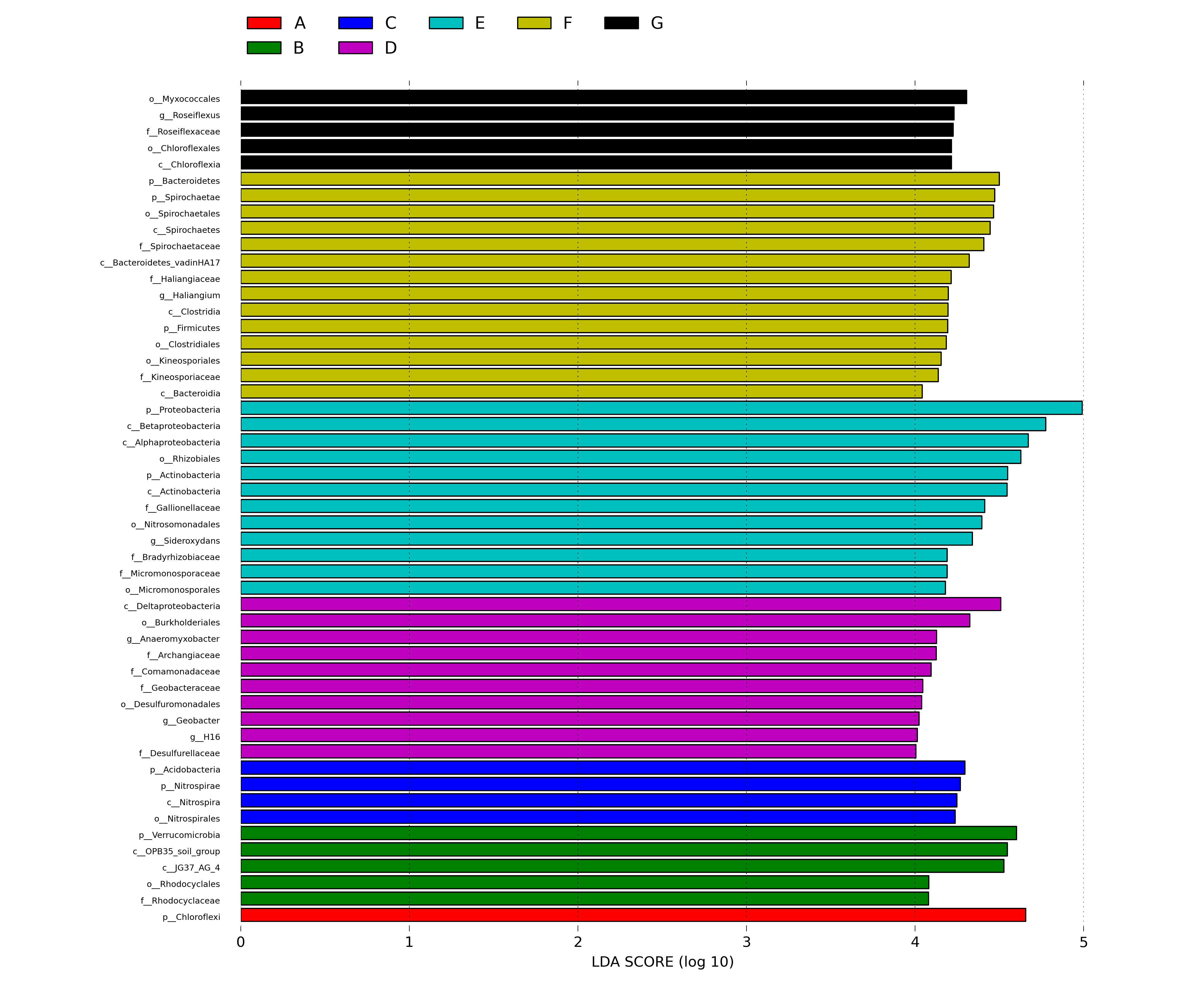

Supplement: Supplementary file 4 — Additional file 4: Fig. S4. Indicator bacterial groups across 8 compartments with LDA values higher than 3. LDA: linear discriminant analysis. A:NPB-Mock-B.R, B: Pizt-Mock-B.R, C: NPB-KJ201-B.R, D: Piz-t-KJ201-B.R; E:NPB-Mock-B.E, F: Pizt-Mock-B.E, G: NPB-KJ201-B.E, H: Piz-t-KJ201-B.E. [file 12284_2021_486_MOESM4_ESM.png]
